# Exploring under-five child malnutrition in Bangladesh: analysis using the Extended Composite Index of Anthropometric Failure (ECIAF)

**DOI:** 10.1017/S1368980025000138

**Published:** 2025-02-03

**Authors:** Farzana Akhter Bornee, Mohammad Rocky Khan Chowdhury, Bodrun Naher Siddiquea, Baki Billah, Farjana Akter, Md Nazmul Karim

**Affiliations:** 1 Department of Pediatrics, Bangabandhu Sheikh Mujib Medical University, Dhaka, Bangladesh; 2 Department of Epidemiology and Preventive Medicine, School of Public Health and Preventive Medicine, Faculty of Medicine, Nursing and Health Sciences, Monash University, Melbourne, Australia; 3 Department of Sociology and Social Work, The People’s University of Bangladesh, Dhaka, Bangladesh

**Keywords:** Bangladesh, Children, Malnutrition, Undernutrition, Overweight, Composite index

## Abstract

**Objective:**

The current study is an attempt to explore under-five child malnutrition in a low-income population setting using the Extended Composite Index of Anthropometric Failure (ECIAF).

**Design:**

Data from the Bangladesh Demographic and Health Survey 2017–2018 were analysed. Malnutrition using ECIAF was estimated using stunting, wasting underweight and overweight. Multilevel logistic regression models identified factors associated with malnutrition. Geospatial analysis was conducted using R programming.

**Setting:**

Bangladesh.

**Participants:**

Children under 5 years of age.

**Results:**

In Bangladesh, as indicated by the ECIAF, approximately 40·8 % (95 % CI: 39·7, 41·9) of children under five experience malnutrition, whereas about 3·3 % (95 % CI: 2·9, 3·7) were overweight. Children of parents with no formal education (56·3 %, 95 % CI: 50·8, 61·8), underweight mothers (53·4 %, 95 % CI: 50·4, 56·3), belonging to the lowest socio-economic strata (50·6 %, 95 % CI: 48·3, 53·0), residing in rural areas (43·3 %, 95 % CI: 41·9, 44·6) and aged below 3 years (47·7 %, 95 % CI: 45·2, 50·2) demonstrated a greater age- and sex-adjusted prevalence of malnutrition. The Sylhet division (Eastern region) exhibited a higher prevalence of malnutrition (> 55·0 %). Mothers with no formal education (adjusted OR (AOR): 1·51, 95 % CI: 1·08, 2·10), underweight mother (AOR: 1·54, 95 % CI: 1·03, 1·83), poorest socio-economic status (AOR: 2·14, 95 % CI: 1·64, 2·81), children aged 24–35 months (AOR: 2·37, 95 % CI: 1·97, 2·85) and fourth and above birth order children (AOR: 1·41, 95 % CI: 1·16, 1·72) were identified key factors associated with childhood malnutrition while adjusting community- and household-level variations.

**Conclusions:**

In Bangladesh, two out of five children were malnourished, and one in thirty-five children was overweight. Continuous monitoring of the ECIAF over time would facilitate tracking changes in the prevalence of different forms of malnutrition, helping to plan interventions and assess the effectiveness of interventions aimed at addressing both undernutrition and overweight.

Child malnutrition, classified as indicators of anthropometric failure (AF) such as stunting, wasting, underweight and overweight, often refers to the inadequate or excessive intake of essential nutrients necessary for the healthy growth and development of children^(1)^. Child malnutrition continues to be a significant public health issue for children under 5 years in many low- and middle-income countries^(2)^. According to current reports, approximately 148·1 million children under five suffer from stunting, 45 million experience wasting and 82 million are underweight worldwide^(2)^. Furthermore, approximately 37 million children in this age group are affected by overweight^(2)^. Undernutrition is responsible for approximately half of all under-five child deaths worldwide^(1)^. Moreover, deaths associated with overweight surpass those related to underweight in the majority of the world’s population^(3)^. A possible reason could be that global estimates of under-five child malnutrition are likely to be underestimated. Additionally, many existing tools for assessing malnutrition primarily focus on undernutrition (i.e. stunting, wasting and underweight), neglecting the opposite end of the spectrum – overnutrition^(4–9)^. The failure to include the entire spectrum has the potential to underestimate the true impact of nutritional disorders, particularly the synergistic effects of the coexistence of multiple forms and the double burden of malnutrition (i.e. stunting, wasting, underweight and overweight). These effects include increased susceptibility to infections, chronic diseases, impaired physical growth and cognitive development, higher healthcare costs and reduced economic productivity^(10)^.

The standard estimates or single indicators often overlap, despite reflecting distinct biological processes. As a result, they are unable to provide an accurate and complete assessment of undernourishment among subjects. For example, some stunted children might also be wasted, while others may simultaneously experience both stunting and being underweight. In some cases, children may suffer from all three conditions concurrently. Consequently, these traditional metrics do not adequately capture the overall burden of malnutrition^(11)^. In nutritional assessment, several composite indices are used. These indices combine multiple indicators of AF, along with their interactions, into a single summary measure, providing a more comprehensive and accurate picture of the nutritional status of a population than any single indicator alone^(12–15)^. Recent literature reveals a multitude of efforts to use composite indices^(16–19)^. However, the majority of these indices were not able to address the ‘elephant in the room’. Specifically, they have overlooked being overweight, which has increasingly become a concern in low-resource settings. According to the WHO, many nations are grappling with the dual burden of malnutrition. This phenomenon highlights the simultaneous presence of inadequate nutrition (e.g. stunting) and excessive weight (e.g. overweight and obesity)^(20,21)^. The prevalence of undernutrition, once prominent in numerous low- and middle-income countries, is now giving way to elevated levels of overweight and obesity. This shift can be attributed to transitions in nutrition, epidemiology and demographics^(10)^.

The composite index proposed by Bejarano *et al*. (2019) adopts a holistic approach by viewing the spectrum of nutritional disorders, from undernutrition to overnutrition, under the umbrella of malnutrition^(21)^. They reported approximately a threefold increase in the prevalence of malnutrition compared with undernutrition when malnutrition was assessed using the proposed index. The use of such tools in the assessment of malnutrition is likely to reveal the true extent of the malnutrition problem in low-income countries like Bangladesh, where policy planning has historically been heavily focused on undernutrition^(22)^. Despite a significant apparent reduction in under-five child undernutrition, the prevalence of child malnutrition, addressing both undernutrition and overnutrition in Bangladesh, is likely underestimated, as these are measured using available indices that primarily focus on undernutrition^(4,12,17)^. This study attempts to explore under-five child malnutrition in Bangladesh using one such tool, the Extended Composite Index of Anthropometric Failure (ECIAF).

## Methods

### Data source: Bangladesh Demographic and Health Survey

We analysed data from the Bangladesh Demographic and Health Survey (BDHS) 2017–2018. The data were collected between 24 October 2017 and 15 March 2018. The survey collected demographic data on Bangladeshi adults, information on maternal health, reproductive health and maternal and child nutritional status from all eight administrative regions (divisions): Barisal (southern region), Chittagong (southeastern region), Dhaka (central region), Khulna (western region), Mymensingh (upper-central region), Rajshahi (midwestern region), Rangpur (northwestern region) and Sylhet (eastern region). The survey adopted a multistage cluster sampling technique. In the first stage, 675 primary sampling units (PSU) were selected using probability proportional to size, with 250 PSU from urban areas and 425 PSU from rural areas. The PSU were based on enumeration areas (clusters) listed in the 2011 population census conducted by the Bangladesh Bureau of Statistics^(23)^. In the second stage, an average of thirty households was selected from each PSU using an equal probability systematic sampling technique. The multistage sampling and corresponding sampling weights were designed to reduce potential sampling bias. Additionally, all ever-married women aged 15–49 years (with or without children under 5 years) from the preselected households were interviewed without replacement or changes in the implementation stage to prevent selection bias. A total of 20 127 women aged 15–49 years were interviewed from 19 457 households, achieving a response rate of 99 %. In BDHS 2017–2018, a total of 8759 children under five were listed, and 7902 children were found eligible for analysis (online Supplementary Figure 1). Detailed methodology is described in the BDHS 2017–2018 report^(23)^.

### Malnutrition assessment

Nandy *et al*. (2005) introduced the concept of the Composite Index of Anthropometric Failure (CIAF) aimed at assessing undernutrition. This index encompassed seven distinct AF groups: (A) absence of failure; (B) only wasting; (C) co-occurrence of wasting and underweight; (D) simultaneous presence of wasting, stunting and underweight; (E) stunting and underweight; (F) only stunting; and (G) only underweight^(16)^. Later, Bejarano *et al*. (2019) expanded upon this concept by introducing ECIAF to gauge child malnutrition. They proposed a broader range of nine AF categories: (A) absence of failure; (B) only wasting; (C) co-occurrence of wasting and underweight; (D) simultaneous presence of wasting, stunting and underweight; (E) stunting and underweight; (F) only stunting; (G) only underweight; (H) only overweight; and (I) co-occurring overweight and stunting^(21,22)^. The ECIAF is computed by subtracting the count of group A children from the total sample, and it served as a tool to measure the outcome of under-five child malnutrition in this study (online Supplementary Table1).

Four commonly used AF measures of child malnutrition are stunting, wasting, underweight and overweight. A child was considered to be stunted (short stature for age), wasted (dangerously thin) and underweight (low weight for age) if the height-for-age, weight-for-height and weight-for-age indices were 2 sd or more below the respective median of the WHO reference population. Conversely, a child was considered to be overweight if the weight-for-height index was 2 sd or more above the respective median of the WHO reference population^(24,25)^.

### Operational definitions of demographic and other characteristics

Parental characteristics, children characteristics, household, home environment and contextual factors that were considered in the study are pooled from relevant literatures^(13,17,19,26,27)^. The parental characteristics include maternal age in years (15–19, 20–24, 25–29, 30–34, 35–39, ≥ 40), parents’ education (both parents uneducated, only father was uneducated when the mother was educated, only mother was uneducated when father was educated, both parents educated), mother’s working status (not working or currently working), mother’s body size (normal, underweight and overweight), mother’s attitudes towards wife beating (justified, not justified); mother’s decision-making autonomy (participated, not participated) and father’s occupational status (currently not working, manual labourer, service holder, businessman). Household factor includes mass media exposure (no, ye) and source of drinking water (improved, unimproved). Home environmental factors are the use of cooking fuel (non-solid, solid) and type of toilet facilities (improved, unimproved). Contextual factors comprise wealth index (poorest, poorer, middle, richest and richer), place of residence (urban, rural) and region of residence (Barisal, Chittagong, Dhaka, Khulna, Mymensingh, Rajshahi, Rangpur, Sylhet). Factors related to children are children’s age (0–11 months, 12–23 months, 24–35 months, 36–47 months and 48–59 months), sex of child (male, female), birth order (first, second, third and fourth and above), diarrhoea (no, yes) and acute respiratory infection (no, yes). Operational definitions of selected factors were presented in online Supplementary Table 3.

### Statistical analysis

Descriptive statistics were used to present the background characteristics of the children. Bivariate analysis (*χ* ^2^ test) was used to explore the significant associations (significance level set at *P* < 0·05) between the independent variables and malnutrition as per the ECIAF among under-five children. Initially, a multivariable multilevel binary logistic regression model with a random intercept term at the community and household level was performed to identify significant determinants of childhood malnutrition. In DHS, individuals are nested within households, and households are nested within communities, which indicates that individuals, households and communities are not independent of each other^(4,28)^. We employed a multilevel random intercept model to account for variation in different levels (community, household and individual levels). Further, multivariable binary logistic regression analysis (fixed effect model) was performed to identify the determinants of malnutrition. Variables that were found to be significant at the level *P* < 0·25 in bivariate analysis were included in the multivariable analysis^(29–32)^. The level of significance for multivariable regression analysis was set at *P* < 0·05, and an OR with 95 % CI was estimated to determine the associated factors. The interclass correlation coefficient was generated out and identified as 25 % for the random effect model, which indicated a low interclass correlation coefficient. Further, the estimated value of Akaike’s information criterion and Bayesian information criterion for the multilevel model was 10 206·2 and 10 408·3 and slightly lower compared with the fixed effect regression model (online Supplementary Table 4). Therefore, the random effect model could be considered as a better model compared with the fixed effect model. Stata version 17 (StataCorp LP) was used for all analyses. To control the effect of the complex survey design, all bivariate and multivariable analyses of this study were performed using Stata’s ‘svyset’ and melogit commands, respectively. Geospatial analysis was carried out using R programming language (4.3.3) to visualise the regional prevalence of malnutrition as per the ECIAF and CIAF and regional prevalence of childhood overweight.

## Results

A significant proportion (62·8 %) of the mothers fell within the younger age bracket (20–29 years). About 7·1 % of the children’s mothers had received no formal education (with 4·0 % having both parents uneducated and 3·1 % being solely uneducated mothers). About 15 % of the mothers were classified as underweight. Approximately 47·8 % of the children were female, and about 40 % of the children were from socio-economically disadvantaged backgrounds, and a substantial majority (65·9 %) of all children were residents of rural areas. For more comprehensive information, please see Table 1, which outlines these background characteristics in detail.

**Table 1.** Background characteristics of respondents

Factors	Number (*n*)	Unweighted percentage (%)	Weighted percentage (%)
Maternal age (in years)			
15–19	985	12·5	13·0
20–24	2753	34·8	34·7
25–29	2210	28·0	28·1
30–34	1326	16·7	16·7
34–39	497	6·3	6·0
≥ 40	131	1·7	1·6
Parents’ education			
Both parents uneducated	318	4·0	4·1
Only father uneducated	987	12·5	12·4
Only mother uneducated	248	3·1	3·1
Both parents educated	6349	80·4	80·4
Mother’s currently working			
No working	4701	59·5	59·6
Currently working	3201	40·5	40·4
Mother’s body size (BMI)			
Normal	4688	59·3	60·3
Underweight	1167	14·8	14·0
Overweight	2047	25·9	25·8
Mother’s attitude towards wife beating			
Not justified	6462	81·8	81·2
Justified	1440	18·2	18·8
Mothers’ decision-making autonomy			
Not practised	1222	15·5	15·2
Practised	6680	84·5	84·8
Fathers’ occupational status			
Currently not working	212	2·7	2·6
Manual labourer	5529	70·0	71·3
Service holder	481	6·1	5·4
Businessman	1680	21·3	20·7
Source of drinking water			
Improved	6867	86·9	86·9
Not improved	1035	13·1	13·1
Use of cooking fuel			
Non-solid	2286	28·9	29·7
Solid	5616	71·1	70·3
Type of toilet facilities			
Improved	4487	56·8	56·8
Not improved	3415	43·2	43·2
Mass media exposure			
No	2863	36·2	35·0
Yes	5039	63·8	65·0
Wealth index			
Poorest	1767	22·4	21·8
Poorer	1597	20·2	20·5
Middle	1430	18·1	19·2
Richer	1565	19·8	20·0
Richest	1543	19·5	18·5
Place of residence			
Urban	2694	34·1	26·4
Rural	5208	65·9	73·6
Children age			
0–11 months	1736	22·0	21·8
12–23 months	1617	20·5	20·7
24–35 months	1530	19·3	19·6
36–47 months	1459	18·5	18·7
48–59 months	1560	19·7	19·2
Sex of child			
Male	4125	52·2	52·3
Female	3777	47·8	47·7
Birth order			
First	3019	38·2	38·2
Second	2573	32·6	32·5
Third	1327	16·8	16·9
Fourth and above	983	12·4	12·4
Diarrhoea prior 2 weeks			
No	7507	95·0	95·2
Yes	395	5·0	4·8
ARI prior 2 weeks			
No	6878	87·0	86·7
Yes	1024	13·0	13·3
Total	7902	100·0	100·0

ARI, acute respiratory infection.

### Prevalence and geospatial distribution of child malnutrition

**Figure 1.**
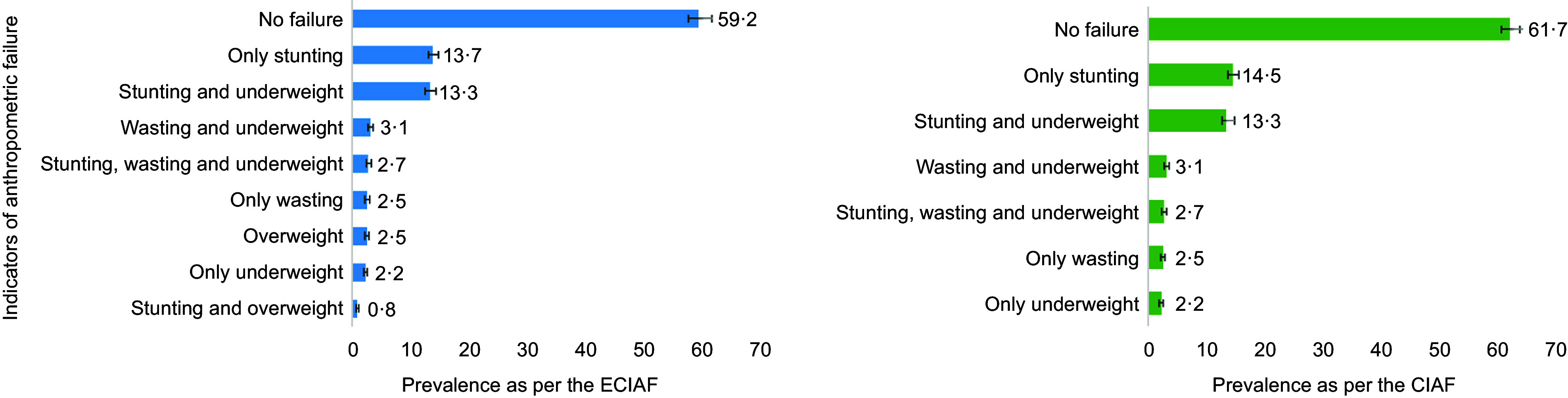
Prevalence of various indicators of anthropometric failures (malnutrition indicators). ECIAF, Extended Composite Index of Anthropometric Failure; CIAF, Composite Index of Anthropometric Failure.

In Bangladesh, as per the ECIAF, the prevalence of under-five child malnutrition was 40·8 % (95 % CI: 39·7, 41·9), while the CIAF indicates that about 38·3 % (95 % CI: 37·2, 39·5) of children experience undernourishment, as illustrated in Fig. 1. Moreover, approximately 3·3 % (95 % CI: 2·9, 3·7) of children face overweight conditions alongside other forms of undernutrition, as highlighted in Fig. 1.

**Figure 2.**
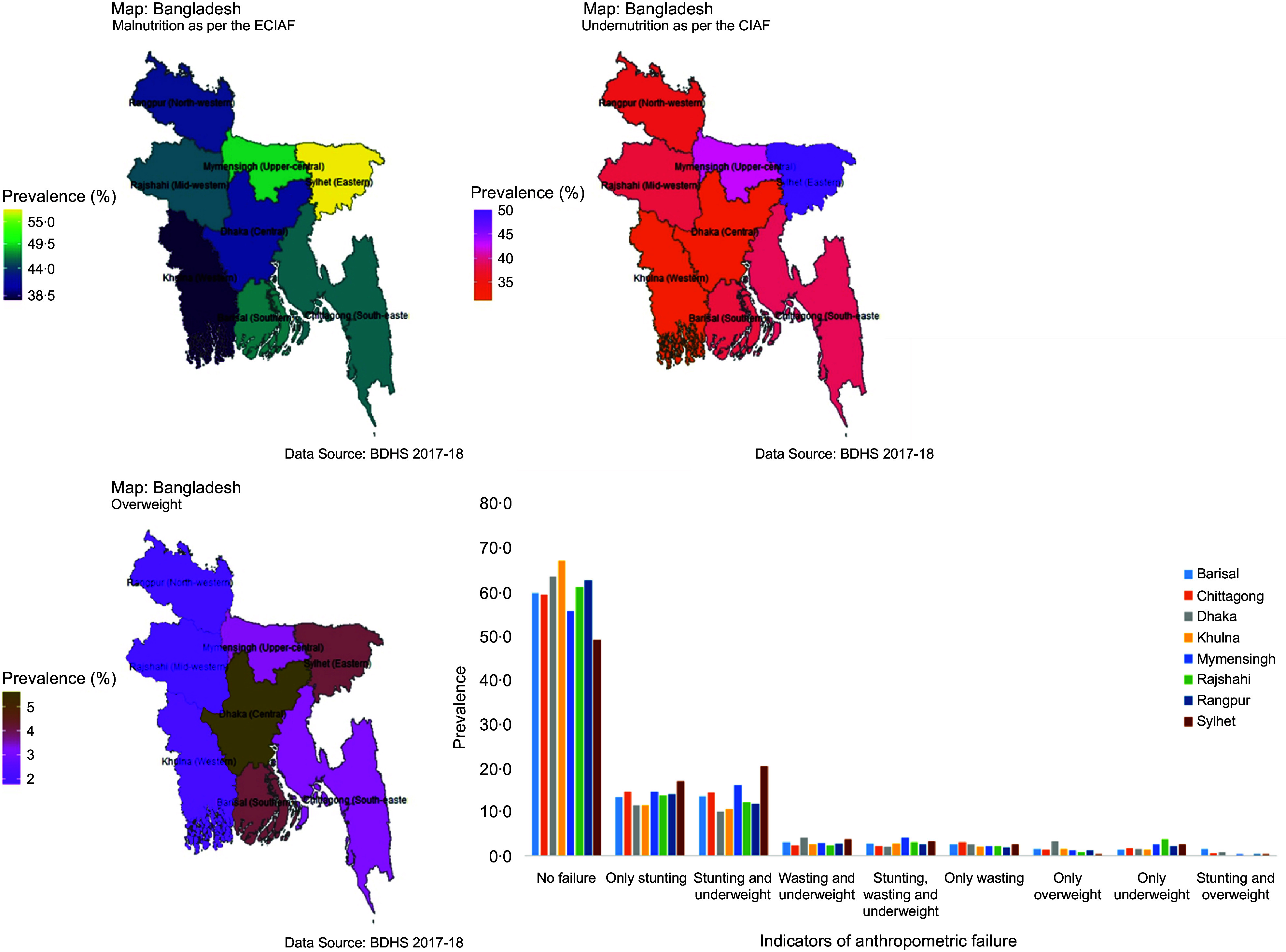
Geospatial distribution of prevalence of malnutrition, undernutrition and overweight. BDHS, Bangladesh Demographic and Health Survey; CIAF, Composite Index of Anthropometric Failure; ECIAF, Extended Composite Index of Anthropometric Failure.

Notably, the prevalence of malnutrition is higher in the Sylhet division (eastern region), reaching over 55 %, compared with the lower prevalence observed in the Khulna division (western region) (below 38 %). Similarly, the prevalence of undernutrition according to the CIAF is also elevated in the Sylhet division (eastern region) (approximately 50 %). On the other hand, the Dhaka division (central region) exhibits a higher prevalence of under-five children overweight (over 5 %), followed by Barisal (southern) and Sylhet (eastern) divisions, each at approximately 4 %, as depicted in Fig. 2.

### The age- and sex-adjusted prevalence of child malnutrition

Among children, the age- and sex-adjusted prevalence of malnutrition was significantly higher at 56·3 % (95 % CI: 50·8, 61·8) in households where mothers had no formal education. Similarly, a heightened prevalence was observed among children born to underweight mothers (53·4 %, 95 % CI: 50·4, 56·3), those in families with a fourth or higher birth order (52·3 %, 95 % CI: 48·1, 54·4), children from the most socio-economically poorest (50·6 %, 95 % CI: 48·3, 53·0) and those aged 24–35 months (47·7 %, 95 % CI: 45·2, 50·2), as detailed in Table 2.

**Table 2.** Prevalence of under-five child malnutrition as per the ECIAF

Factors	Number (*n*)	Crude prevalence	95 % CI	Age- and sex-adjusted prevalence	95 % CI	*P*-values
Overall	3262	40·6	39·3, 42·4	40·8	39·7, 41·9	
Maternal age (in years)						
15–19	420	41·2	37·6, 44·9	44·0	40·8, 47·2	0·927
20–24	1097	40·1	37·7, 42·5	39·6	37·8, 41·5	
25–29	912	41·0	38·6, 43·5	41·0	38·9, 43·0	
30–34	559	41·3	38·1, 44·6	41·6	38·9, 44·2	
34–39	213	41·0	36·1, 46·1	42·6	38·2, 46·9	
≥ 40	61	45·4	35·6, 55·5	45·4	36·9, 54·0	
Parents’ education						
Both parents uneducated	180	55·8	49·3, 62·1	56·3	50·8, 61·8	< 0·001
Only father uneducated	490	49·9	46·4, 53·5	49·6	46·4, 52·7	
Only mother uneducated	133	51·6	44·2, 59·0	53·9	47·7, 60·1	
Both parents educated	2459	38·3	36·7, 39·8	38·6	37·4, 39·8	
Mother’s working status						
No	1891	39·6	37·7, 41·6	40·3	39·0, 41·8	0·034
Yes	1371	42·6	40·5, 44·8	42·3	40·6, 44·0	
Mother’s body size						
Normal	1959	41·2	39·2, 43·1	43·3	41·6, 45·0	< 0·001
Underweight	621	51·6	48·4, 54·7	53·4	50·4, 56·3	
Overweight	682	34·2	31·8, 36·8	34·5	32·9, 36·2	
Mother’s attitude towards wife beating						
Not justified	2640	40·3	38·7, 41·9	40·7	39·5, 41·9	0·096
Justified	622	43·0	40·0, 46·1	43·1	40·6, 45·7	
Mothers’ decision-making autonomy						
Not practised	518	41·2	38·1, 44·3	42·3	39·5, 45·0	0·824
Practised	2744	40·8	39·1, 42·4	40·9	39·8, 42·1	
Fathers’ occupational status						
Currently not working	96	45·6	38·3, 53·2	45·0	38·2, 51·7	< 0·001
Manual labourer	2417	43·3	41·6, 45·0	43·6	42·3, 44·9	
Service holder	133	26·4	22·4, 30·8	27·6	23·5, 31·6	
Businessman	616	35·6	32·8, 38·4	36·4	34·1, 38·7	
Source of drinking water						
Improved	2848	41·1	39·5, 42·7	41·0	40·0, 42·4	0·225
Not improved	414	39·0	35·7, 42·3	40·8	37·7, 43·8	
Use of cooking fuel						
Non-solid	807	36·0	33·4, 38·7	35·5	33·5, 37·4	< 0·001
Solid	2455	42·9	41·1, 44·6	43·4	42·2, 44·8	
Type of toilet facilities						
Improved	1718	38·1	36·1, 40·1	38·0	36·6, 39·4	< 0·001
Not improved	1544	44·4	42·4, 46·5	45·3	43·6, 47·0	
Mass media exposure						
No	1352	46·4	44·2, 48·8	47·4	45·5, 49·2	< 0·001
Yes	1910	37·8	36·0, 39·7	37·6	36·3, 39·0	
Wealth index						
Poorest	897	50·0	47·2, 52·8	50·6	48·3, 53·0	< 0·001
Poorer	753	45·6	42·5, 48·6	47·2	44·7, 49·7	
Middle	565	38·3	35·4, 41·3	39·4	36·9, 42·0	
Richer	606	39·0	36·2, 42·0	38·7	36·2, 41·1	
Richest	441	29·4	26·4, 32·6	28·2	25·9, 30·40	
Place of residence						
Urban	1003	37·2	34·4, 40·1	37·1	35·2, 38·9	0·005
Rural	2259	42·1	40·3, 43·9	43·3	41·9, 44·6	
Children age						
0–11 months	563	32·0	29·5, 34·7	32·5	30·3, 34·7	< 0·001
12–23 months	713	42·6	39·8, 45·6	43·9	41·5, 46·3	
24–35 months	728	47·8	44·7, 50·8	47·7	45·2, 50·2	
36–47 months	645	43·6	40·6, 46·7	44·1	41·5, 46·6	
48–59 months	613	39·1	36·3, 42·0	39·3	36·9, 41·7	
Sex of child						
Male	1731	41·8	39·9, 43·7	41·8	40·3, 43·3	0·119
Female	1531	39·8	37·8, 41·8	40·4	38·8, 42·0	
Birth order						
First	1177	39·0	37·0, 41·2	38·7	36·9, 40·0	< 0·001
Second	1013	39·3	36·9, 41·7	39·4	37·5, 41·3	
Third	570	41·4	38·4, 44·5	42·8	40·0, 45·4	
Fourth and above	502	49·6	45·7, 53·6	51·3	48·1, 54·4	
Diarrhoea prior 2 weeks						
No	3110	40·9	39·4, 42·5	41·3	40·2, 42·4	0·536
Yes	395	39·1	33·6, 44·9	38·2	33·3, 43·0	
ARI prior 2 weeks						
No	2833	40·8	39·2, 42·4	41·0	39·8, 42·2	0·924
Yes	429	41·0	37·7, 44·3	42·3	39·2, 45·3	

ARI, acute respiratory infection; CI, confidence interval.

### Factors associated with child malnutrition

Table 3 reveals that simple logistic regression analysis yielded significant associations between under-five child malnutrition and various factors, including parental education, mother currently working, mother’s body size, use of cooking fuel, type of toilet facilities, mass media exposure, wealth index, place of residence, children age and birth order.

**Table 3.** Determinants of under-five child malnutrition as per the ECIAF

Factors	COR	95 % CI	*P*-values	AORr	95 % CI	*P*-values
Parents’ education						
Both parents uneducated	1·88	1·53, 2·29	< 0·001	1·43	1·05, 1·95	0·021
Only father uneducated	1·57	1·37, 1·82	< 0·001	1·28	1·07, 1·54	0·008
Only mother uneducated	1·83	1·41, 2·36	< 0·001	1·51	1·08, 2·10	0·016
Both parents educated (RC)	1·00			1·00		
Mother’s working status						
No	1·00			1·00		
Yes	1·11	1·02, 1·22	0·021	0·91	0·81, 1·03	0·156
Mother’s body size						
Normal (RC)	1·00			1·00		
Underweight	1·58	1·39, 1·80	< 0·001	1·54	1·30, 1·83	< 0·001
Overweight	0·70	0·62, 0·77	< 0·001	0·79	0·69, 0·90	< 0·001
Mother’s attitude towards wife beating						
Not justified (RC)	1·00			1·00		
Justified	1·10	0·98, 1·23	0·103	0·99	0·86, 1·15	0·942
Fathers’ occupational status						
Currently not working	1·43	1·07, 1·91	0·015	1·14	0·79, 1·67	0·476
Manual labourer	1·34	1·20, 1·50	< 0·001	1·17	1·01, 1·36	0·031
Service holder	0·66	0·52, 0·82	< 0·001	0·88	0·66, 1·17	0·386
Businessman (RC)	1·00			1·00		
Use of cooking fuel						
Non-solid (RC)	1·00			1·00		
Solid	1·42	1·29, 1·57	< 0·001	0·97	0·83, 1·14	0·750
Type of toilet facilities						
Improved (RC)	1·00			1·00		
Not improved	1·33	1·21, 1·45	< 0·001	1·05	0·92, 1·21	0·425
Mass media exposure						
No	1·46	1·33, 1·61	< 0·001	1·04	0·90, 1·20	0·584
Yes (RC)	1·00			1·00		
Wealth index						
Poorest	2·58	2·23, 2·98	< 0·001	2·14	1·64, 2·81	< 0·001
Poorer	2·23	1·92, 2·58	< 0·001	1·98	1·54, 2·54	< 0·001
Middle	1·63	1·40, 1·90	< 0·001	1·52	1·21, 1·91	< 0·001
Richer	1·58	1·36, 1·83	< 0·001	1·56	1·26, 1·92	< 0·001
Richest (RC)	1·00			1·00		
Place of residence						
Urban (RC)	1·00			1·00		
Rural	1·29	1·17, 1·42	< 0·001	0·98	0·84, 1·15	0·864
Children age						
0–11 months (RC)	1·00			1·00		
12–23 months	0·61	0·53, 0·70	< 0·001	1·89	1·58, 2·25	< 0·001
24–35 months	1·16	1·01, 1·34	0·032	2·37	1·97, 2·85	< 0·001
36–47 months	1·01	0·87, 1·16	0·918	2·05	1·70, 2·46	< 0·001
48–59 months	0·82	0·71, 0·95	0·008	1·55	1·29, 1·85	< 0·001
Sex of child						
Male (RC)	1·00			1·00		
Female	0·94	0·86, 1·03	0·192	0·93	0·83, 1·04	0·192
Birth order						
First (RC)	1·00			1·00		
Second	1·02	0·91, 1·13	0·769	1·06	0·92, 1·21	0·416
Third	1·19	1·03, 1·34	0·014	1·15	0·97, 1·36	0·100
Fourth and above	1·63	1·41, 1·89	< 0·001	1·41	1·16, 1·72	0·001

AORr, adjusted odds ratio-random; COR, crude odds ratio; CI, confidence interval; RC, reference category.

For the adjusted model, the random effect model was preferred over the fixed effect model to find out the association between outcome and exposures (Table 3). The national level data consist of multiple entities (e.g. states, regions, households) where each entity is assumed to have its unique and uncorrelated characteristics. The random effect model focuses on understanding how the effects of explanatory variables differ across various entities and aims to estimate entity-specific variances, whereas the fixed effect model assumes unobserved entity-specific effects that are correlated with the explanatory variables.

In Table 3, children had a higher likelihood of being malnourished in mothers with no formal education (adjusted OR-random (AORr): 1·51, 95 % CI: 1·08, 2·10)) compared with children with both parents educated. Further, children of underweight mothers (AORr: 1·54, 95 %: 1·03, 1·83), fathers being manual labourer (AORr: 1·17, 95 % CI: 1·01, 1·36), poorest socio-economic status (AORr: 2·14, 95 % CI: 1·64, 2·81), children age 24–35 months of age (AORr: 2·37, 95 % CI: 1·97, 2·85) and fourth and above birth order children (AORr: 1·41, 95 % CI: 1·16, 1·72) had significant effects on under-five child malnutrition when considering community- and household-level variations. In comparison, the findings of the fixed effect model were presented in online Supplementary Table 4.

In the sensitivity analysis, a multilevel logistic regression model (random effects model) was utilised, incorporating four additional variables into the primary model. These variables were minimum acceptable diet, low birth weight, antenatal care and prenatal care, focused on children aged 6–23 months (online Supplementary Table 5). The analysis revealed that children born with low birth weight had a higher likelihood of malnutrition (AORr: 2·16, 95 % CI: 1·29, 3·59) compared with children of normal birth weight.

## Discussion

While the CIAF was utilised to provide a comprehensive view of the undernutrition status among children under the age of five in Bangladesh, revealing a prevalence of 38·3 %, the ECIAF was employed to broaden the analysis by including overweight prevalence (3·3 %). This resulted in a more inclusive measurement of malnutrition, with a prevalence of 40·8 %. This approach offers a more thorough evaluation of the nutritional well-being of children, spanning the entire spectrum from undernutrition to overnutrition. Although undernutrition rates in Bangladesh have been declining, the gradual rise in childhood overweight poses a significant concern. The prevalence of childhood overweight in the country increased from 1·6 % in 2004 to 2·3 % in 2014 and further escalated to 3 % in 2019^(33)^. A comparative analysis across South Asian nations indicates that Bangladesh’s childhood overweight rate (3·3 %) is lower than that of Maldives (5·4 %) and Pakistan (4·9 %) and yet surpasses that of India (2·8 %) and Nepal (1·4 %)^(34)^.

Among all the administrative divisions in Bangladesh, the Sylhet region (located in the eastern hilly part) exhibited the highest prevalence of malnutrition as indicated by the ECIAF, mirroring the pattern observed for undernutrition based on the CIAF. These findings align with the results of prior studies conducted in Bangladesh^(4,17)^. The Sylhet region includes the Haor basin, characterised by its distinctive wetland landscape resembling an oxbow lake. This terrain necessitates specific agricultural approaches^(35)^. Residents of various Haor areas frequently experience food insecurity due to sudden pre-monsoon floods and the reliance on cultivating only one crop. Additionally, the region faces pronounced education- and wealth-based inequalities^(36)^. While child undernutrition was prevalent among economically disadvantaged and less-educated parents, issues of childhood overweight were observed among children from more affluent backgrounds in the same area. To address these challenges, strategies to reduce poverty in the most vulnerable communities could include measures such as enhancing employment opportunities, ensuring a stable food supply and improving female literacy rates. At the same time, strengthening awareness programmes across all social strata could help mitigate childhood malnutrition^(37–39)^. The current study revealed that the prevalence of overweight in children under five was highest in Dhaka (located in the central plain region) at 5·6 %, followed by Barisal (southern region) at 4·2 % and Sylhet (eastern region) at 4·2 %. Dhaka, being a central region, faces rapid urbanisation, which has led to limited play spaces for children^(40)^. This scarcity contributes to inadequate physical activity, increased screen time, unhealthy dietary patterns and greater access to processed and calorie-dense foods. These factors collectively drive the rising incidence of childhood overweight.

This study also found that malnutrition was highly prevalent among children of uneducated parents, underweight mothers and children of fourth or higher birth order. Furthermore, mothers with no formal education, underweight mothers, poorest socio-economic status, children aged 24–35 months and children of fourth or higher birth order were identified as key factors associated with childhood malnutrition, while accounting for community- and household-level variations. Nonetheless, comparable factors exhibited significance in relation to malnutrition, albeit with varying odds ratios when analysed using a fixed-effects model. These crucial determinants have been previously established as significant contributors to childhood undernutrition in numerous studies conducted in Bangladesh and other developing regions^(13,15,17,26,27)^. It is worth noting that these past investigations employed a fixed-effects model, unlike ours, which may have underestimated the variations present at different levels or failed to account for potential heterogeneity among groups, such as urban and rural children.

Previous studies in Bangladesh focused on under-five child undernutrition have highlighted various causative factors. For instance, the condition of uneducated and impoverished women often leads to maternal underweight, which consequently contributes to child undernutrition^(4,12)^. Additionally, inappropriate and insufficient initiation of complementary feeding, as well as the prioritisation of older children within a household when multiple children are present, can adversely affect the nutritional status of younger children^(41)^. These studies have proposed potential solutions to address undernutrition, including strategies such as enhancing educational attainment, improving access to health and nutrition services, ensuring proper maternal healthcare utilisation to improve maternal health, implementing effective interventions targeted at vulnerable groups (particularly children from the most socio-economically disadvantaged backgrounds), fostering parental engagement through awareness initiatives, implementing multisectoral healthcare programmes to reduce the prevalence of undernutrition and reinforcing family planning efforts to delay childbirth and reduce higher-order births^(4,5,12,13,17,41)^. However, the government of Bangladesh and other relevant stakeholders have already undertaken many undernutrition alleviation strategies, such as the National Adaptation Plan of Bangladesh (2023–2050), Meeting the Undernutrition Challenge (MUCH) and the National Food and Nutrition Security Policy Plan of Action (2021–2030), among others^(42–44)^. Despite these concerted efforts, child undernutrition remains high in Bangladesh. A lack of multisectoral planning and coordination (involving government, non-governmental, social, cultural and religious institutions), gaps in food and nutrition information and the absence of quality epidemiological data are some of the complex reasons preventing significant improvements in the nutritional status of children in Bangladesh^(45,46)^.

### Future direction and policy implication

To address childhood malnutrition effectively, it is crucial to establish and monitor cross-sectoral coordination (involving government, non-governmental, social, cultural and religious institutions). Ensuring proper maternal nutrition before, during and after pregnancy is essential for a child’s health. The focus should be directed towards the critical first thousand days of a child’s life, promoting nutritious, varied and safe dietary practices from the outset to prevent both undernutrition and obesity^(47)^. This includes fostering a healthy environment with access to essential health services, clean water, proper sanitation and opportunities for safe physical activity. Improving living conditions and enhancing maternal nutritional knowledge can significantly reduce rates of childhood malnutrition^(48)^. Elevating the status of women should also be a priority. To tackle both undernourishment and overnutrition, authorities must enhance the quantity and quality of available food while ensuring it remains affordable. Community-level education programmes and public awareness campaigns are vital for encouraging healthy lifestyles and dietary habits. Further research, particularly among preschool children in rural areas and across different ethnic groups, should be conducted using the ECIAF model to better understand the scope of malnutrition. The ECIAF can provide valuable epidemiological information for monitoring the progress of public health policies^(21,22)^. Considering the findings from current investigations, including the distribution of childhood malnutrition, education and social status and family planning efforts to delay childbirth and reduce higher-order births, can inform the development of improved health and nutritional policies. Additionally, reshaping existing strategies and interventions using the double-duty action proposed by Hawkes *et al*. (2020) may help address the double burden of malnutrition^(49)^. Last but not least, policies, programmes and resources cannot be successfully adopted, implemented or sustained without strong commitment.

### Limitation of using the ECIAF

The ECIAF, which typically aggregates multiple indicators into a single value, may not always be the best tool for diagnosing the nutritional status of individuals. By combining different malnutrition indicators such as stunting, wasting, underweight and overweight into a single measure, the composite index may overly simplify the complexities of nutritional issues, potentially obscuring specific problems like micronutrient deficiencies. People have unique health conditions, genetic predispositions and lifestyle factors that influence their nutritional needs. A composite index standardised for a general population might not accurately reflect these individual differences. Composite indices are often designed to capture broad trends rather than diagnose specific health conditions. They may not be sensitive enough to detect mild or emerging nutritional problems in an individual. When indicators are combined, high values in one indicator can offset low values in another, potentially creating a misleading picture of an individual’s health. This can lead to underestimation of risk or need in specific nutritional areas. Nutritional factors also differ widely across cultural and regional contexts, and a universal index might not accurately capture local dietary practices, economic conditions or health priorities, thereby limiting its effectiveness.

Composite indices are often used in research and policy-making to provide a general overview of health or nutritional status across populations or groups. They simplify complex data, making it easier to communicate and make broad comparisons. However, heavy reliance on a single composite index for policy-making could skew directions and resource allocations, overlooking crucial aspects necessary to tackle the root causes of malnutrition. To overcome these limitations, it is essential to adopt more nuanced approaches that integrate local contexts and specific nutritional issues into health assessments and interventions.

### Strength and limitation of the study

The large sample size and robust statistical techniques are key strengths of the study. Furthermore, this is a comprehensive assessment of under-five child malnutrition, covering both undernutrition and overnutrition, using nationally representative samples. One limitation of the study is that the cross-sectional nature of the data does not allow for the determination of causal relationships. The data were collected retrospectively and were self-reported; thus, there is a possibility of underreporting and recall bias. In addition, other important variables, such as complementary feeding practices, antenatal care, postnatal care and birth weight, were not adjusted for due to a large number of missing observations. Nutrient intake and micronutrient status could not be adjusted in the model due to a lack of data.

### Conclusion

The findings revealed that two out of five children in Bangladesh were malnourished, with a higher prevalence in the eastern region (Sylhet division) and a higher prevalence of overweight in the central region (Dhaka division). Children born to mothers with no formal education, underweight mothers, those from the poorest socio-economic backgrounds, children aged 24–35 months and those born as the fourth child or above are at greater risk of developing malnutrition. Despite its limited occurrence in Bangladesh, the inclusion of overweight was considered a significant component in the composite index, designed as the ECIAF, to assess the comprehensive state of malnutrition.

Continuous monitoring of the ECIAF over time has the potential to facilitate tracking changes in the prevalence of different forms of malnutrition, helping to assess the effectiveness of interventions aimed at addressing both undernutrition and overweight. Further research on composite indices is important to enhance their reliability and applicability.

## Supporting information

Bornee et al. supplementary material Bornee et al. supplementary material

## Supplementary material

For supplementary material accompanying this paper, visit https://doi.org/10.1017/S1368980025000138.
