# Supplementary material for: Exploring under-five child malnutrition in Bangladesh: analysis using the Extended Composite Index of Anthropometric Failure (ECIAF)
Source: Public Health Nutr. 2025 Feb 3;28(1):e43. doi: 10.1017/S1368980025000138 (PMC11883563; doi:10.1017/S1368980025000138)
Supplement: Bornee et al. supplementary material [file S1368980025000138sup001.docx]

**Supplementary**

A total of 8,759 children were recorded in BDHS 2017-18

375 children were excluded due to death

8,402 children were selected for height and weight measurement

500 children were excluded for not taking height and weight measurements

Finally, 7,902 children were selected for analysis

**Figure 1** Schematic presentation of sample size selection

**Table 1** Classification of children with severe anthropometric failure

| **Group name** | **Description** | **Wasting** | **Stunting** | **Underweight** | **Overweight** |
| --- | --- | --- | --- | --- | --- |
| A | No failure | No | No | No | No |
| B | Only wasting | Yes | No | No | No |
| C | Wasting and underweight | Yes | No | Yes | No |
| D | Wasting, stunting and underweight | Yes | Yes | Yes | No |
| E | Stunting and underweight | No | Yes | Yes | No |
| F | Only stunting | No | Yes | No | No |
| G | Only underweight | No | No | Yes | No |
| H | Only overweight | No | No | No | Yes |
| I | Overweight and stunting | No | Yes | No | Yes |

**Table 2** Measurements of independent variables

| **Variable** | **Description** | **Measurement** | **Scale of measurement** | **Number of responses/Missing** |
| --- | --- | --- | --- | --- |
| **Maternal age (in years)** | Age of mothers at the time of data collection | 15-19, 20-24, 25-29, 30-34, 35-39, ≥ 40 | Categorial | 7,902/0 |
| **Parents’ education** | The educational status of parents is determined by whether they have completed a minimum of five years of schooling, which corresponds to the primary level encompassing classes 1 to 5, indicating that they are considered educated. On the other hand, if they have received no formal education or have completed zero years of schooling, they are classified as uneducated | Both parents were uneducated, only father was uneducated, only mother was uneducated, both parents were educated | Categorical | 7,902/0 |
| **Mother’s working status** | Was the mother actively involved in economic pursuits during the data collection period? | Not working, currently working | Binary | 7,902/0 |
| **Mother’s body size** | A mother having a body mass index (BMI) less than 18.5 kg/m² is categorized as underweight, while a BMI within the range of 18.5 to 24.9 kg/m² is considered normal, and a BMI equal to or greater than 25 kg/m² is classified as overweight. | Normal, underweight, overweight | Binary | 7,902/0 |
| **Mother’s attitudes toward wife-beating** | Respondents are queried about their agreement with the notion that a husband is justified in hitting or beating his wife in the following five situations: when she burns the food, when she engages in arguments with him, when she goes out without informing him, when she neglects the children, and when she declines to engage in sexual activity with him. If respondents respond affirmatively to at least one of these situations, they are categorized as having attitudes attitudes justifying wife beating. | Not justified, justified | Binary | 7,902/0 |
| **Mothers’ decision-making autonomy** | In the BDHS surveys, a woman's decision-making authority is evaluated based on three main criteria: 1) a woman who usually decides on her healthcare 2) a woman who usually decides on large household purchases and 3) a woman who usually decides on visits to family or relatives. The available response options included: (a) respondent alone, (b) respondent and husband/partner, (c) respondent and another person, (d) husband/partner alone, (e) someone else, (f) other. For each of these questions, a score of 0 was assigned to indicate a lack of decision-making ability if the response was d, e, or f. Otherwise, a score of 1 was given for responses a, b, or c. These individual scores were then summed, resulting in a total score ranging from 0 to 3. The Cronbach’s α for the instruments was 0·81, which indicates a high level of internal consistency. | Not participated, participated | Binary | 7,902/0 |
| **Father’s occupational status** | The current workforce can be categorized as follows: "Currently not working" includes individuals who are unemployed or are currently students. "Manual labourers" comprise farmers, agricultural workers, fishermen, rickshaw drivers, and others engaged in physically demanding occupations. "Service holders" encompass professionals like doctors, lawyers, accountants, and teachers. "Businessman" engages in various business activities, including both small and large-scale enterprises. | Currently not working, manual labourers, service holders and businessman | Categorical | 7,902/0 |
| **Mass media exposure** | Mass media exposure, such as television, radio, newspapers, or magazines, is considered to occur when an individual is exposed to at least one of these media sources at least once a week. | No, Yes | Binary | 7,902/0 |
| **Source of drinking water** | Improved sources of water supply can be categorized as follows: piped water within the dwelling, piped water to the yard or plot, access to a public tap or standpipe, piped water from a neighboring source, tube wells or boreholes, protected wells, protected springs, rainwater harvesting, tanker truck delivery, carts with small tanks, and bottled water. Unimproved sources of water supply, on the other hand, include: unprotected wells, unprotected springs, surface water from sources such as rivers, dams, lakes, ponds, streams, canals, and irrigation channels, as well as any other sources not falling within the improved category. | Improved, unimproved | Binary | 7,902/0 |
| **Use of cooking fuel** | Solid fuel encompasses substances like coal, lignite, charcoal, wood, straw, shrubs, grass, agricultural crops, and animal dung. Non-solid fuel comprises electricity, natural gas, processed gas, biogas, and kerosene. | Non-solid fuel, Solid fuel | Binary | 7,902/0 |
| **Type of toilet facility** | Improved sanitation facilities can be categorized as follows: flush toilets connected to a piped sewer system, flush toilets connected to a septic tank, flush toilets connected to a pit latrine, flush toilets with an unspecified disposal system, ventilated improved pit latrines, pit latrines with a slab, and composting toilets.  Unimproved sanitation facilities include: flush toilets with disposal to an unspecified location, pit latrines without a slab or in open pits, bucket toilets, hanging toilets/latrines, and other non-specified options. | Improved, unimproved | Binary | 7,902/0 |
| **Wealth index** | In DHS surveys, the wealth index is determined by the DHS authority through a calculation that takes into account household characteristics and assets using principal component analysis. Subsequently, households are divided into five categories or quintiles based on their wealth index scores, with those having lower index values categorized as the poorest, and those with higher values as richest. | Poorest, poorer, middle, richer, richest | Categorical | 7,902/0 |
| **Place of residence** | Place of residence | Different residential areas across the country. | Urban, rural | 7,902/0 |
| **Region of residence** | Administrative regions of Bangladesh | Barisal, Chittagong, Dhaka, Khulna, Mymensingh, Rajshahi, Rangpur, Sylhet | Categorical | 7,902/0 |
| **Children’s age (in months)** | Age of the children at the time of data collection | 0-11 months, 12-23 months, 24-35 months, 36-47 months, 48-59 months | Categorical | 7,902/0 |
| **Sex of child** | Sex differential of children | Male, female | Binary | 7,902/0 |
| **Birth order** | Birth order is the chronological order of sibling births in a family | One, two, three, four and above | Categorical | 7,902/0 |
| **Diarrhea** | Children who had diarrhea in the 2 weeks preceding the survey and received oral rehydration solution (ORS), and advice or treatment from health facilities or qualified health providers | No, yes | Binary | 7,902/0 |
| **Acute respiratory infection (ARI)** | Children had symptoms of ARI (short, rapid breathing which was chest-related, and/or difficult breathing which was chest-related) in the 2 weeks preceding the survey and received advice or treatment from health facilities or qualified health providers. | No, yes | Binary | 7,902/0 |

**Table 3** Random effect and fixed effect matrices.

| **Models** | **Matrices** | |
| --- | --- | --- |
| **Random effect model** |  |  |
| Level 3: Community variance | 0.93 (0.23*) | 0.93 (0.23*) |
| Level 2: Household Variance | 0.15 (0.04*) | 0.15 (0.04*) |
| ICC (Community \| Household) | 0.25 (0.04) | 0.25 (0.04) |
| AIC | 10206.19 | 10206.19 |
| BIC | 10408.31 | 10408.31 |
| **Fixed effect model** |  |  |
| AIC |  |  |
| BIC |  |  |

AIC, Akaike's information criterion, BIC, Bayesian information criterion, ICC, Interclass correlation coefficient

**‘∗’** denotes the standard error (SE) of random intercept and it measures the variability of average effect in each level (community and household) to experience malnutrition. The *P* value for each random effect variance is 0.118.

**Table 4** Results of fixed effect model

| **Factors** | **Fixed effect model** | |
| --- | --- | --- |
|  | **AORf (95% CI)** | **p-values** |
| **Parents’ education** |  |  |
| Both parents uneducated | 1.38 (1.08, 1.76) | 0.010 |
| Only father uneducated | 1.23 (1.07, 1.43) | 0.004 |
| Only mother uneducated | 1.42 (1.09, 1.84) | 0.010 |
| Both parents educated | 1.00 |  |
| **Mother’s working status** |  |  |
| No | 1.00 |  |
| Yes | 0.89 (0.81, 0.98) | 0.030 |
| **Mother’s body size** |  |  |
| Normal | 1.00 |  |
| Underweight | 1.41 (1.23, 1.61) | <0.001 |
| Overweight | 0.80 (0.72, 0.89) | <0.001 |
| **Mother’s attitude towards inmate partner violence** |  |  |
| Not Justified | 1.00 |  |
| Justified | 0.99 (0.88, 1.12) | 0.956 |
| **Fathers’ occupational status** |  |  |
| Currently not working | 1.13 (0.83, 1.54) | 0.412 |
| Manual labourer | 1.14 (1.01, 1.28) | 0.028 |
| Service holder | 0.89 (0.70, 1.12) | 0.330 |
| Businessman | 1.00 |  |
| **Use of cooking fuel** |  |  |
| Non-solid | 1.00 |  |
| Solid | 0.98 (0.86, 1.11) | 0.788 |
| **Type of toilet facilities** |  |  |
| Improved | 1.00 |  |
| Not improved | 1.04 (0.93, 1.15) | 0.446 |
| **Mass media exposure** |  |  |
| No | 1.05 (0.94, 1.17) | 0.375 |
| Yes | 1.00 |  |
| **Wealth index** |  |  |
| Poorest | 1.85 (1.50, 2.27) | <0.001 |
| Poorer | 1.77 (1.46, 2.15) | <0.001 |
| Middle | 1.41 (1.18, 1.69) | <0.001 |
| Richer | 1.44 (1.22, 1.70) | <0.001 |
| Richest | 1.00 |  |
| **Place of residence** |  |  |
| Urban | 1.00 |  |
| Rural | 0.98 (0.88, 1.09) | 0.728 |
| **Children age** |  |  |
| 0-11 months | 1.00 |  |
| 12-23 months | 1.63 (1.42, 1.89) | <0.001 |
| 24-35 months | 2.01 (1.73, 2.33) | <0.001 |
| 36-47 months | 1.76 (1.51, 2.04) | <0.001 |
| 48-59 months | 1.40 (1.20, 1.62) | <0.001 |
| **Sex of child** |  |  |
| Male | 1.00 |  |
| Female | 0.94 (0.85, 1.02) | 0.173 |
| **Birth order** |  |  |
| First | 1.00 |  |
| Second | 1.05 (0.94, 1.17) | 0.399 |
| Third | 1.14 (0.99, 1.31) | 0.068 |
| Fourth and above | 1.38 (1.17, 1.61) | <0.001 |

AORf, Adjusted odds ratio-fixed, CI, Confidence interval

**Table 5** Results of random effect model (for children age 6-23 months, n= 2,334)

| **Factors** | **Random effect model** | |
| --- | --- | --- |
|  | **AORr (95% CI)** | **p-values** |
| **Parents’ education** |  |  |
| Both parents uneducated | 1.21 (0.56, 2.25) | 0.748 |
| Only father uneducated | 1.01 (0.42, 2.47) | 0.973 |
| Only mother uneducated | 0.76 (0.39, 1.48) | 0.421 |
| Both parents educated | 1.00 |  |
| **Mother’s working status** |  |  |
| No | 1.00 |  |
| Yes | 0.79 (0.61, 0.1.01) | 0.065 |
| **Mother’s body size** |  |  |
| Normal | 1.00 |  |
| Underweight | 1.83 (1.26, 2.64) | 0.001 |
| Overweight | 0.96 (0.72, 1.28) | 0.809 |
| Obesity | 1.29 (0.81, 2.03) | 0.279 |
| **Mother’s attitude towards inmate partner violence** |  |  |
| Not Justified | 1.00 |  |
| Justified | 1.24 (0.92, 1.68) | 0.140 |
| **Fathers’ occupational status** |  |  |
| Currently not working | 1.00 |  |
| Manual labourer | 1.55 (0.66, 3.63) | 0.313 |
| Service holder | 0.84 (0.31, 2.28) | 0.742 |
| Businessman | 1.22 (0.52, 2.92) | 0.642 |
| **Use of cooking fuel** |  |  |
| Non-solid | 1.00 |  |
| Solid | 1.05 (0.72, 1.52) | 0.798 |
| **Type of toilet facilities** |  |  |
| Improved | 1.00 |  |
| Not improved | 0.96 (0.73, 1.25) | 0.766 |
| **Mass media exposure** |  |  |
| No | 0.97 (0.74, 1.28) | 0.873 |
| Yes | 1.00 |  |
| **Wealth index** |  |  |
| Poorest | 1.00 |  |
| Poorer | 0.98 (0.69, 1.39) | 0.927 |
| Middle | 0.66 (0.44, 1.01) | 0.056 |
| Richer | 0.67 (0.43, 1.05) | 0.082 |
| Richest | 0.46 (0.25, 0.86) | 0.009 |
| **Place of residence** |  |  |
| Urban | 1.00 |  |
| Rural | 0.83 (0.63, 1.11) | 0.232 |
| **Children age** |  |  |
| <12 months | 1.00 |  |
| 12-23 months | 2.04 (1.46, 2.85) | <0.001 |
| **Sex of child** |  |  |
| Male | 1.00 |  |
| Female | 0.84 (0.67, 1.06) | 0.149 |
| **Birth order** |  |  |
| First | 1.00 |  |
| Second | 0.91 (0.70, 1.20) | 0.535 |
| Third | 1.07 (0.75, 1.49) | 0.711 |
| Fourth and above | 1.04 (0.70, 1.53) | 0.839 |
| **Minimum acceptable diet** |  |  |
| No | 1.00 |  |
| Yes | 1.06 (0.84, 1.36) | 0.590 |
| **Antenatal care** |  |  |
| No | 1.00 |  |
| Yes | 1.00 (0.66, 1.52) | 0.995 |
| **Postnatal care** |  |  |
| No | 1.00 |  |
| Yes | 0.88 (0.66, 1.19) | 0.995 |
| **Low birth weight** |  |  |
| No | 1.00 |  |
| Yes | 2.15 (1.28, 3.58) | 0.003 |
| Level 3: Community variance | 0.14 (0.12*) |  |
| Level 2: Household Variance | 1.31 (1.33*) |  |
| ICC (Community \| Household) | 0.31 (2.00*) |  |
| AIC | 3000.96 |  |
| BIC | 3190.88 |  |

AORr, Adjusted odds ratio-random, AIC, Akaike's information criterion, BIC, Bayesian information criterion, CI, Confidence interval, ICC, Interclass correlation coefficient

**‘∗’** denotes the standard error (SE) of random intercept and it measures the variability of average effect in each level (community and household) to experience malnutrition. The *P* value for each random effect variance is 0.118.
